# Research Trends and Hotspots on Montessori Intervention in Patients With Dementia From 2000 to 2021: A Bibliometric Analysis

**DOI:** 10.3389/fpsyt.2021.737270

**Published:** 2021-09-09

**Authors:** Ting Zhou, Jiling Qu, Huiping Sun, Mengxin Xue, Yijing Shen, Yongbing Liu

**Affiliations:** School of Nursing and Public Health, Yangzhou University, Yangzhou, China

**Keywords:** dementia, montessori, research hotspots, CiteSpace, bibliometric analysis, nursing

## Abstract

**Background:** Patients with dementia experience a variety of neuropsychiatric symptoms and behavioral disturbances. The Montessori method is a type of non-pharmacological intervention to care for people with dementia. However, there are few bibliometric studies on the application of Montessori methods. We aimed to analyze the hotspots and trends of research on the application of Montessori methods to the care of dementia patients.

**Methods:** Microsoft Office Excel, Co-Occurrence 9.9, and CiteSpace were used to analyze the articles on Montessori intervention in patients with dementia from 2000 to 2021 in China National Knowledge Infrastructure, Wanfang, China Science and Technology Journal Database, Web of Science core collection database, PubMed, and Scopus.

**Results:** A total of 23 Chinese language publications and 113 English language publications were included. The number of English language publications was on the rise, while the number of Chinese language publications was low. There are many issuing institutions which published articles in this field, mostly concentrated in universities. English language publication sources were more than Chinese language publication sources. The hot research topic in Chinese language publications and English language publications was the care of agitated behavior of dementia patients based on the Montessori method. The psychological problems of dementia patients are likely to become a hot issue of concern for scholars in Chinese. There will be a lot of research focusing on dementia patients and their family caregivers in this field.

**Conclusion:** The bibliometric and visualization analysis helps us understand the current research status and hotspots of Montessori intervention in dementia patients in Chinese language publications and English language publications.

## Introduction

According to a survey, about 50 million people worldwide were living with dementia in 2018 (1). The number of people with dementia is expected to increase to 152 million by 2050 (1), and the number of people with Alzheimer's disease will also exceed 100 million (2). Patients with Alzheimer's disease experience deterioration in cognitive and memory functions, progressive declines in activities of daily living, and a variety of neuropsychiatric symptoms and behavioral disturbances. Behavioral and psychological symptoms of dementia (BPSD) are common symptoms of dementia and manifest as agitation, aggressive behavior, anxiety, and confusion (3). The occurrence of BPSD can cause distress and discomfort for people with dementia, leading to a reduced quality of life (4), as well as increasing the stress and burden on caregivers, making care more costly (5). There are two types of treatments used to reduce BPSD in patients with dementia: pharmacological interventions and non-pharmacological interventions. However, current pharmacological treatments still have major limitations in improving patients' quality of life, and the use of pharmacological interventions can have side effects and increase mortality in patients with dementia (6, 7). It has been reported that two patients with dementia experienced deterioration in their psychiatric behavioral problems while taking direct oral anticoagulants apixaban and rivaroxaban (8). Therefore, scientific and effective nursing interventions are particularly important in the care of patients with dementia.

Non-pharmacological interventions were more effective than pharmacological interventions in reducing BPSD (9). Non-pharmacological interventions, for example, music therapy (10, 11), exercise therapy (12, 13) and aromatherapy (14, 15) can delay or improve patients' cognitive functioning. However, the effectiveness of these non-pharmacological interventions in managing patients' psychiatric behavioral problems is inconsistent (16, 17). However, most patients are passively involved in activities which mentioned above. It is very important for patients to freely participate in activities and learning.

The Montessori approach is a person-centered, non-pharmacological intervention. It is a pedagogical method created by the Italian educator Maria Montessori, which was first applied to children with intellectual disabilities to improve their general abilities by exercising their large muscles and fine motor movements of the hands (18). The approach was later applied for the first time in the field of dementia patient care by the American medical scientist Camp et al. (19). The Montessori approach has three core elements, including a prepared environment, activity materials, and a facilitator. The Montessori approach emphasizes encouraging dementia patients to participate in meaningful activities that match their existing abilities in a prepared environment. The patient's interest and motivation are stimulated through their choice of teaching aids. Through the guidance and encouragement of the guider, patients can exercise their thinking skills and rebuild self-esteem during the activity (20). Compared with other non-pharmacological interventions, the Montessori approach is scientifically based and has been used for over 100 years. The fundamental principle of Montessori approach is to value a patients' naturally inquisitive and improving symptoms of dementia to foster learning in an intuitive and natural way. It was reported that a Montessori-based activity program reduced problem behaviors and improved their quality of life in residents with late-stage dementia (21). In China, a study of 46 dementia patients found that patients who received an individualized Montessori-based activity intervention had significantly reduced frequency and disruptiveness of their verbal aggressive, physical non-aggressive, and physical aggressive behaviors were significantly reduced (22). Currently, several studies have been conducted in China and abroad to investigate the effectiveness of the Montessori approach in dementia patients. However, there are few reports on the research status and hotspots in this field.

Bibliometrics was first proposed by British intelligence scientist Pritchard (23). Bibliometrics is a quantitative analysis method based on various characteristics of literature, including the number of articles, publishing years, authors, institutions, and keywords. It uses mathematical and statistical methods to reveal the current situation and trend of research in a certain field (24). It has also been widely used in the field of nursing research (25, 26).

The CiteSpace software was developed by Dr. Chaomei Chen at Drexel University and runs under a Java environment (27). It is an information visualization software that analyzes a large number of relevant literature data such as authors, keywords, institutions, countries, subject categories and common networks, and predicts new trends in a certain research field and demonstrates new trends. It has been widely used in medicine, information science and other fields (28, 29). Therefore, this study aimed to analyze the published research on the Montessori method applied to the care of dementia patients to understand the current status, hotspots, and development trends in this field.

## Materials and Methods

### Data Sources

A search of articles was conducted in China National Knowledge Infrastructure (CNKI), Wanfang, China Science and Technology Journal Database (VIP) using the subject terms “Montessori” and “dementia.” Inclusion criteria were: (1) Chinese language; (2) literature type of article; and (3) publication year from January 1, 2000 to February 28, 2021. Articles that did not meet the inclusion criteria, as well as conferences, announcements, and other publications, were excluded. A total of 46 articles were retrieved, and 23 were de-duplicated. Save the exported data in refworks format. A search of articles was conducted in Web of Science Core Collection database, PubMed and Scopus with “Montessori” and “dementia” as the subject terms. Inclusion criteria were: (1) English language; (2) literature type of article; and (3) publication year from January 1, 2000 to February 28, 2021. Articles that did not meet the inclusion criteria, as well as conferences, announcements, and other publications, were excluded. A total of 208 articles were retrieved from the Web of Science Core Collection, PubMed and Scopus, and 95 articles were de-duplicated. Save the exported data in refworks format. Two researchers independently screened the articles according to the study inclusion criteria and exclusion criteria, and finally cross-checked. When disagreements were encountered, a third researcher participated in the discussion and made the decision.

### Data Analysis

Microsoft Office Excel 2019, Co-Occurrence 9.9 (30) and CiteSpace 5.7.R2 were used to organize and analyze the included articles. Excel was used to make line charts. Co-Occurrence 9.9 (COOC) is a software package developed by Chinese scholars for bibliometric and scientific graphs. In this study, Co-Occurrence 9.9 was used to extract data from downloaded Chinese and English literature and form documents in an Excel-compatible format. We used Microsoft Office Excel 2019 and COOC to conduct metrological statistics for Chinese language publications and English language publications. Then, Convert the format of the Chinese language publications in CiteSpace, and the converted data can be recognized and analyzed by CiteSpace. The format of English language publications does not need to be converted. Create two new projects for importing and exporting data, respectively. CiteSpace was used to plot the related graphs with nodes of country of publication, institution, keywords, and source. The parameters of CiteSpace were set as follows: time from January 2000 to February 2021; year per slice for 1 year/2 years. Other parameters were default values. CiteSpace automatically generated a visualization plot based on the selection of nodes, and we adjusted the nodes in the visualization window.

## Results

### Annual Distribution of Articles and Country Distribution

**Figure 1:**
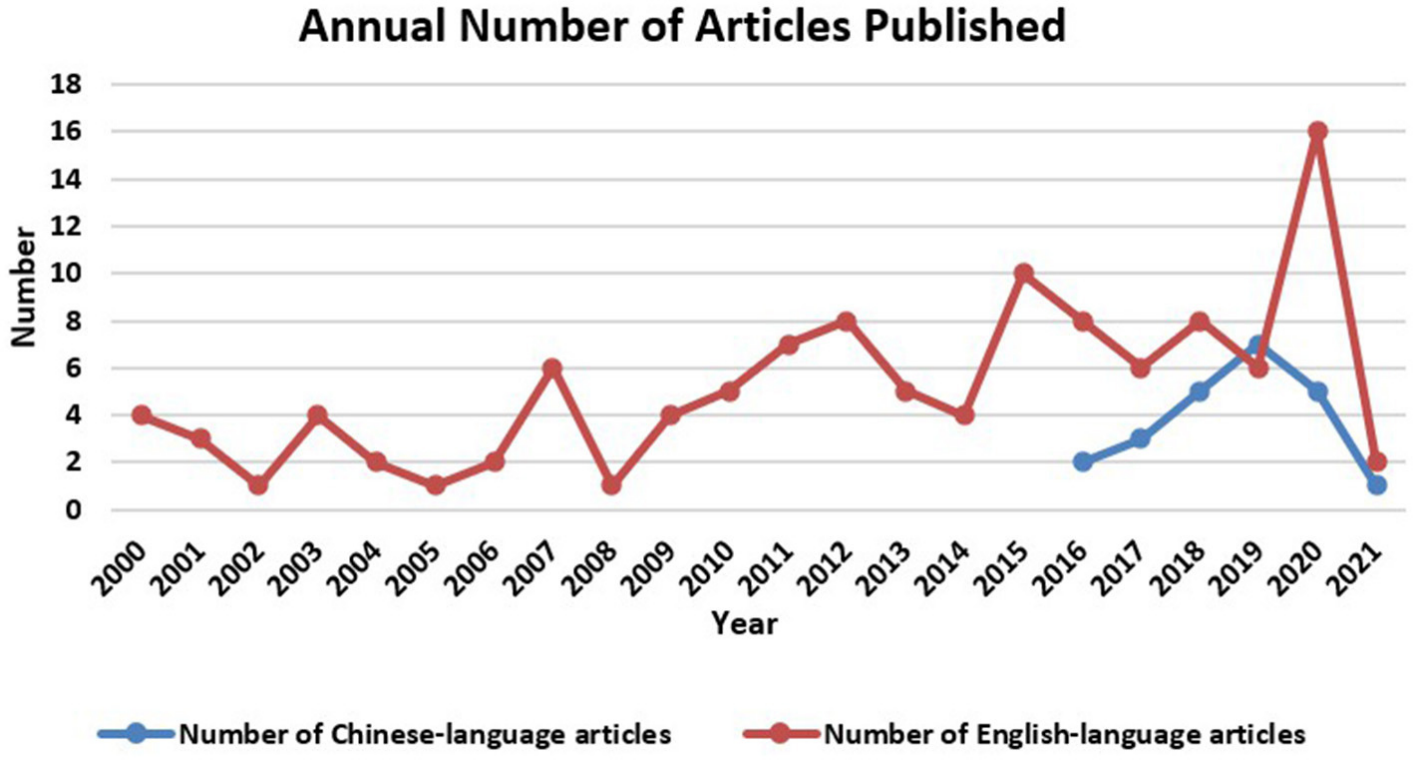
Annual number of articles published in Chinese and English on the study of Montessori intervention in dementia patients from 2000 to 2021.

**Figure 2:**
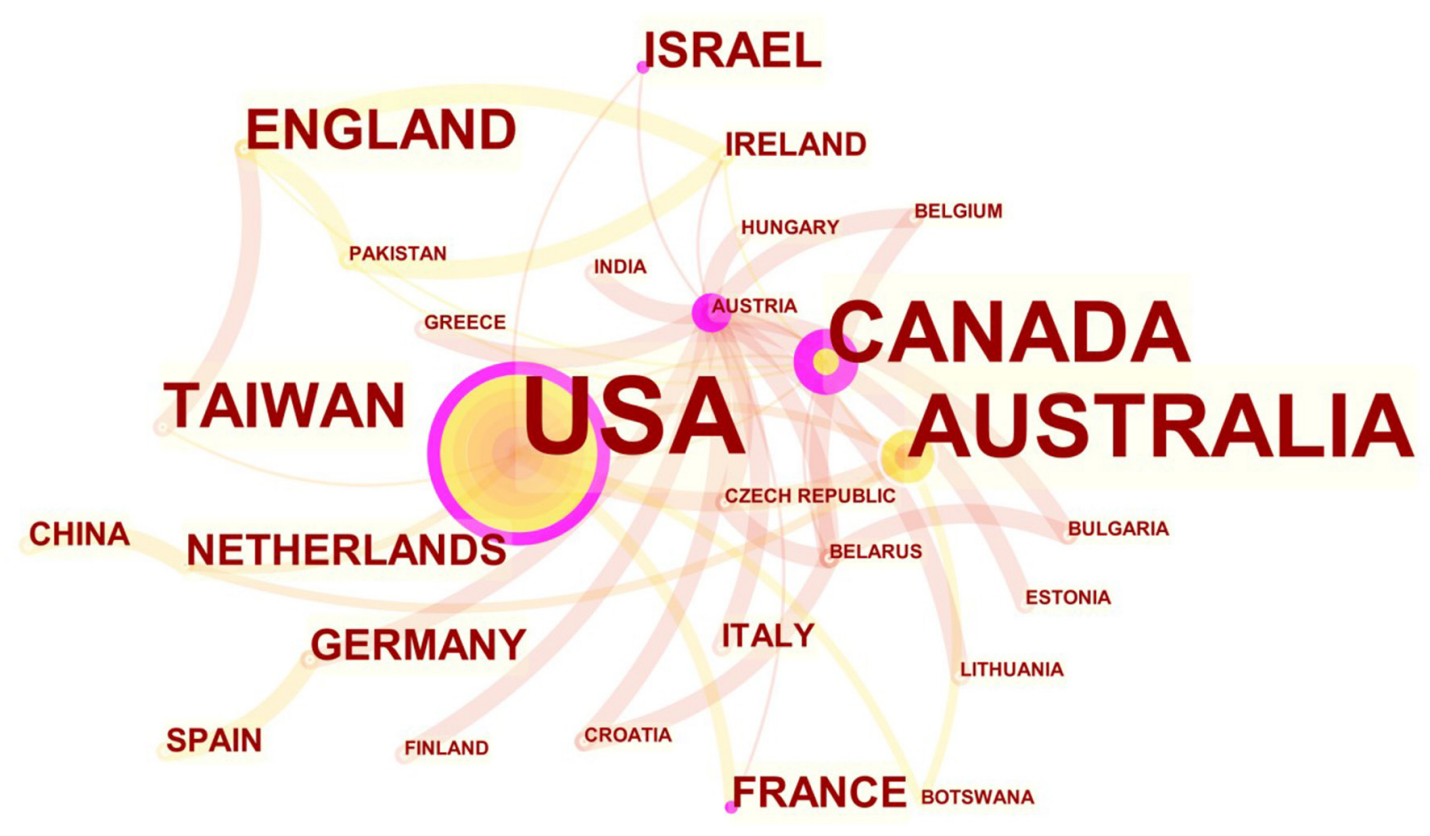
Distribution of national postings in English-language articles on Montessori approach to interventions for patients with dementia from 2000 to 2021.

Figure 1 shows the annual postings of the Chinese language publications and English language publications using Microsoft Office Excel as well as COOC software. There is an overall upward trend in the annual volume of articles written in English on Montessori method interventions for patients with dementia, but the overall volume is low. The number of publications varied considerably from year to year, with the highest number of English language publications in 2020 (16 articles) and fewer in 2002 (1 article), 2005 (1 article), and 2008 (1 article). In contrast, the Chinese language publications on Montessori method interventions for patients with dementia had fewer publications overall, with the highest number of publications in 2019 (7 articles). Figure 2 shows the country distribution of English language publications on Montessori method interventions for dementia patients from 2000 to 2021 using CiteSpace and selecting the Country node for production. A node in the graph represents a country/region, and the larger the node, the higher the number of publications in that country/region. The line between nodes indicates the cooperation between countries/regions. The top three countries in terms of overall publication volume are USA (59 articles), Canada (18 articles), and Australia (17 articles). This indicates that these countries are leaders in research on the effects of Montessori methods of intervention for patients with dementia.

### Distribution of Issuing Institutions

**Figure 3:**
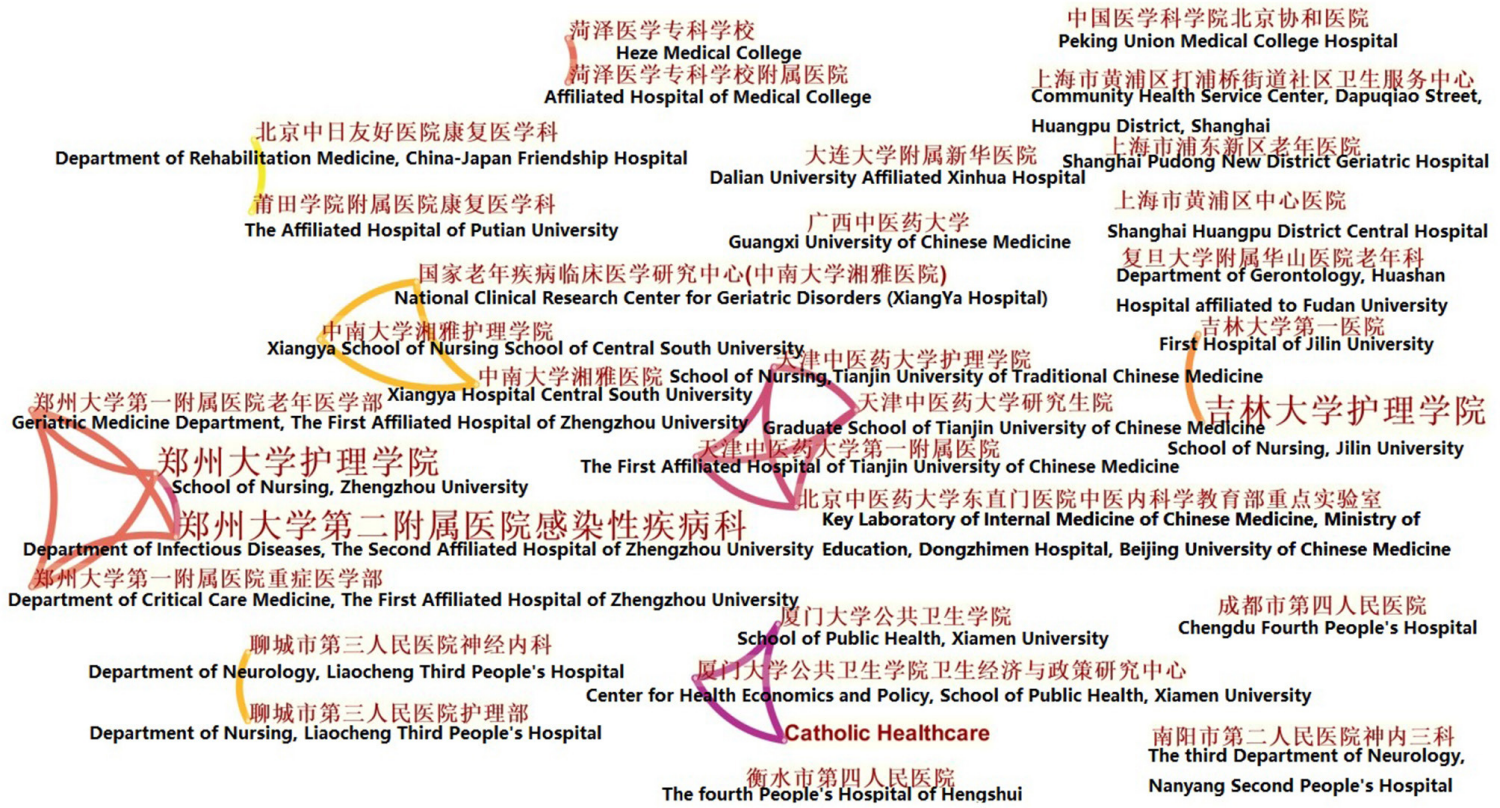
Distribution of institutional publications in the Chinese-language on Montessori approach to interventions for patients with dementia from 2000 to 2021.

**Figure 4:**
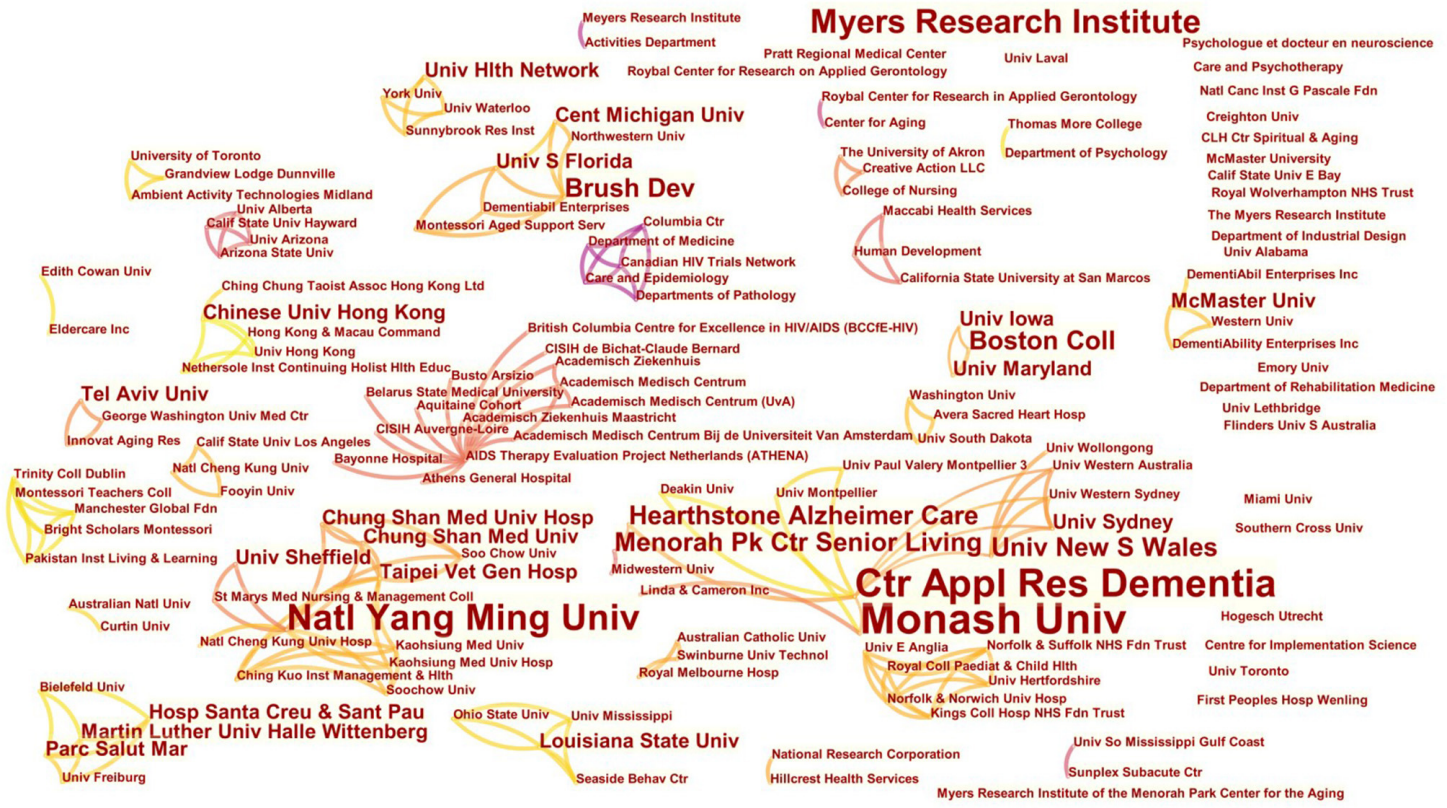
Distribution of institutional publications in the English-language on Montessori approach to interventions for patients with dementia from 2000 to 2021.

Figures 3, 4 show the institutions network mapping of Chinese language publications and English language publications using CiteSpace and selecting the Institution node, where the nodes represent issuing institutions and the connecting lines between nodes represent inter-institutional collaboration. The Chinese-language articles were analyzed, and Figure 3 shows that the top three institutions in terms of the number of publications are the School of Nursing of Zhengzhou University (2 articles), the Second Affiliated Hospital of Zhengzhou University (2 articles), and the School of Nursing of Jilin University (2 articles), while the remaining institutions have one publication. There are institutions in China that have conducted collaborative research with international institutions. However, the distribution of institutions is relatively scattered, and there are fewer collaborative exchanges. Figure 4, an analysis of the English-language articles, shows that the top three institutions in terms of number of publications are Monash University (9 articles), Center for Applied Research in Dementia (7 articles), and National Yang-Ming University (6 articles), and there are more exchanges and cooperation between institutions, forming many cooperative networks.

### Keyword Co-occurrence

**Figure 5:**
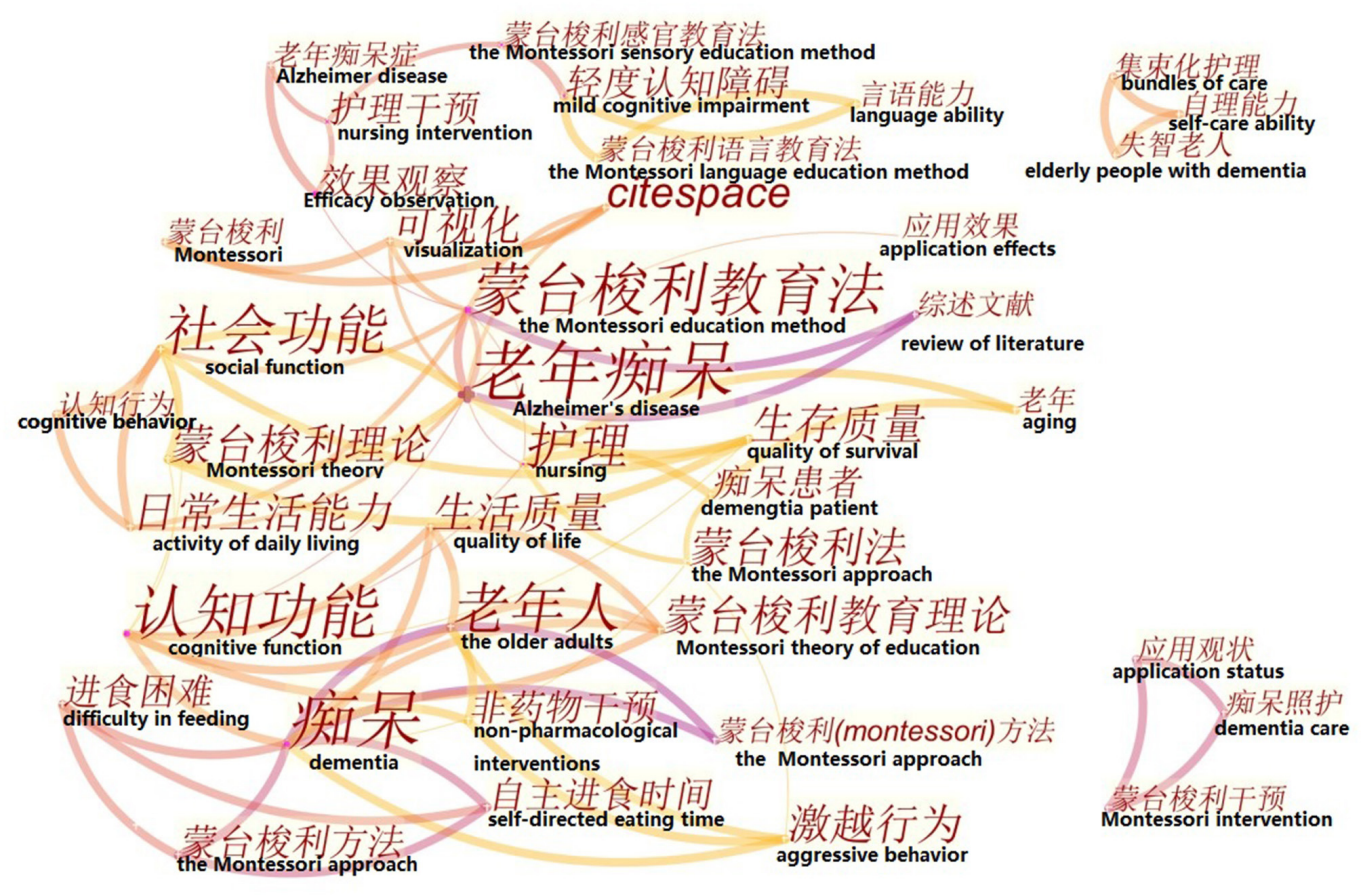
Keyword co-occurrence graph of articles published in Chinese on Montessori approach to interventions for patients with dementia from 2000 to 2021.

**Figure 6:**
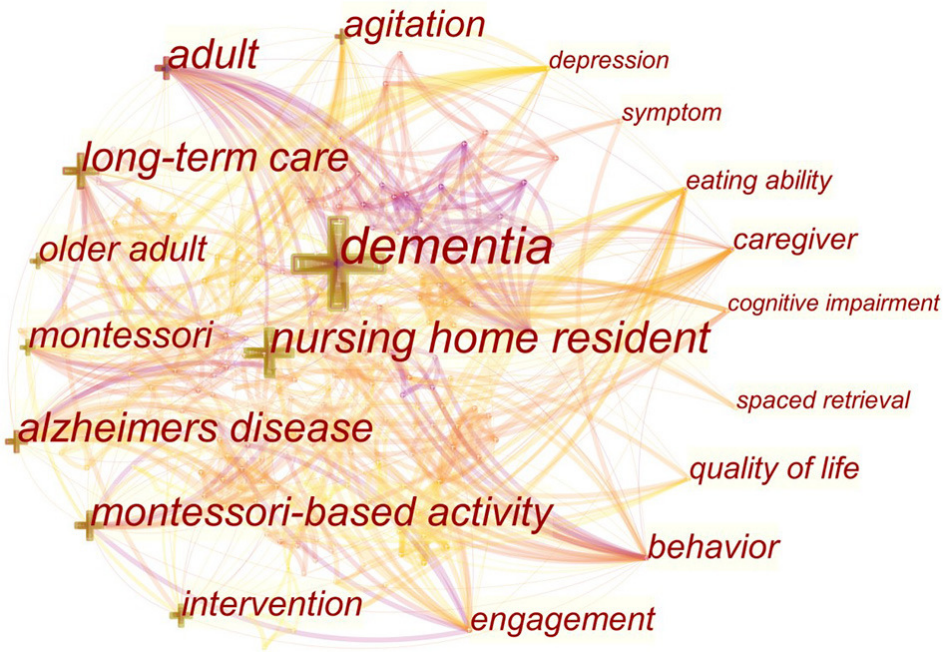
Keyword co-occurrence graph of articles published in English on Montessori approach to interventions for patients with dementia from 2000 to 2021.

In CiteSpace, keywords were used as nodes for analysis. The larger the node in the graph, the higher the frequency of keyword co-occurrences. In Figure 5, the keywords with higher frequency of co-occurrence were “Alzheimer's disease” (6 times), “Montessori pedagogy” (6 times), “dementia” (5 times), “nursing” (4 times), “cognitive function” (3 times), and “elderly” (3 times). The keywords with high co-occurrence centrality were “Montessori education method” (0.34), “cognitive function” (0.32), “Alzheimer's disease” (0.29), “effect observation” (0.27), and “dementia” (0.22). Combining the frequency and centrality of keyword co-occurrence, it can be seen that the hot keywords in Chinese language publications were “Alzheimer's disease,” “Montessori pedagogy,” and “cognitive function.” In Figure 6, the larger the cross-shaped node, the higher the frequency of keyword co-occurrences. The keywords with higher frequency were “dementia” (70 times), “nursing home resident” (35 times), “Alzheimer's disease” (28 times), “adult” (28 times), “long-term care” (27 times), “Montessori-based activity” (26 times), and “agitation” (19 times). The keywords with high centrality of co-occurrence were “adult” (0.23), “Alzheimer's disease” (0.17), “behavior” (0.17), “caregiver” (0.14), “long-term care” (0.13), and “agitation” (0.13). Combining the frequency and centrality of keyword co-occurrence, the hot keywords in English language publications were “Alzheimer's disease,” “adult,” “long-term care,” and “agitation.”

### Keyword Burstness

**Figure 7:**
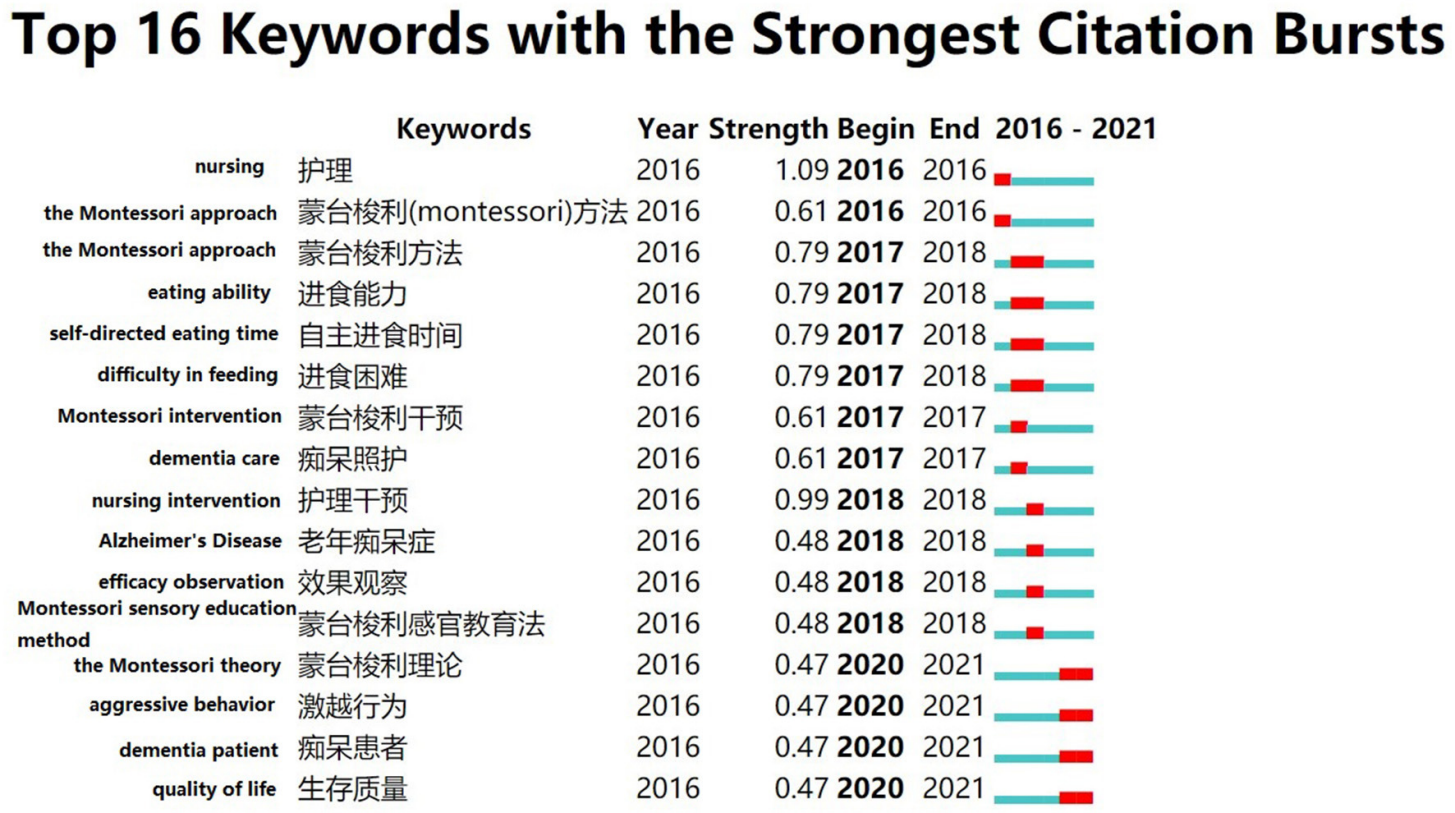
Keyword burst graph of articles published in Chinese on Montessori approach to interventions for dementia patients from 2000 to 2021.

**Figure 8:**
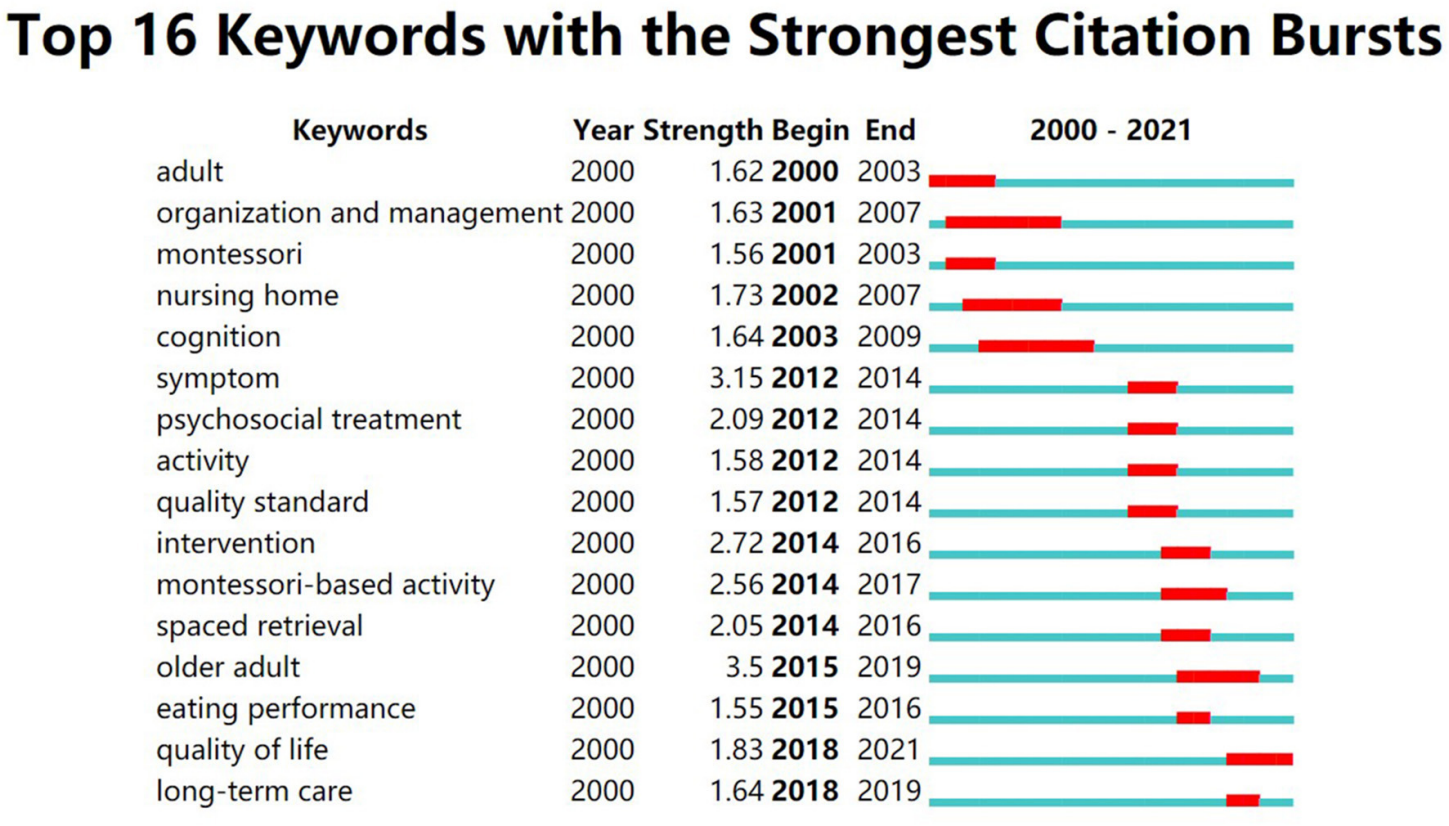
Keyword burst graph of articles published in English on Montessori approach to interventions for patients with dementia from 2000 to 2021.

In CiteSpace, keywords were used as nodes for analysis, and the View function of the Burstness interface was selected for keyword emergence analysis. The emergent analysis of keywords enables the discovery of research hotspots in a field of emergence and when the emergence starts and ends. In Figure 7, the keywords of “time to eat autonomously,” “Montessori method,” “eating difficulties,” “eating ability,” “Montessori theory,” “agonistic behavior,” “dementia patients,” and “quality of survival” have a long burst time of 2 years. Since 2016, China has focused more on intervention studies using the Montessori approach to eating behavior in patients with dementia, while recently the use of the Montessori approach to intervene in the agonistic behavior of patients with dementia is a hot topic of research. In Figure 8, the keywords with longer emergence time are “organization and management,” “nursing home,” “cognition,” “older adult,” “Montessori-based activity,” and “quality of life.” Keyword “quality of life” began to emerge in 2018 and has continued until now, and may also become a hot keyword for research in the future period both domestically and internationally.

### Keywords Timezone Graph

**Figure 9:**
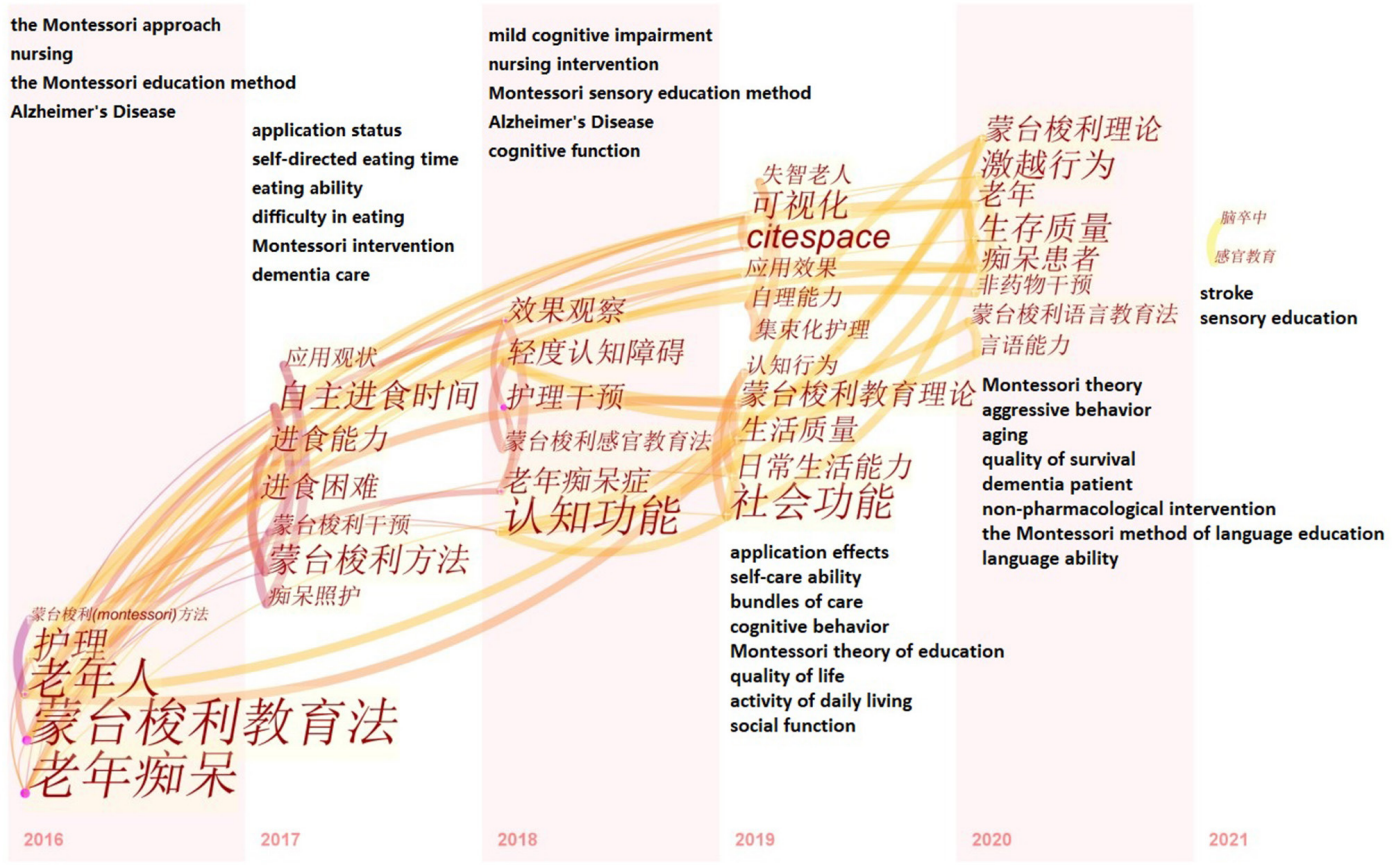
Time zone graph of keywords in articles published in Chinese on Montessori approach to interventions for patients with dementia from 2000 to 2021.

**Figure 10:**
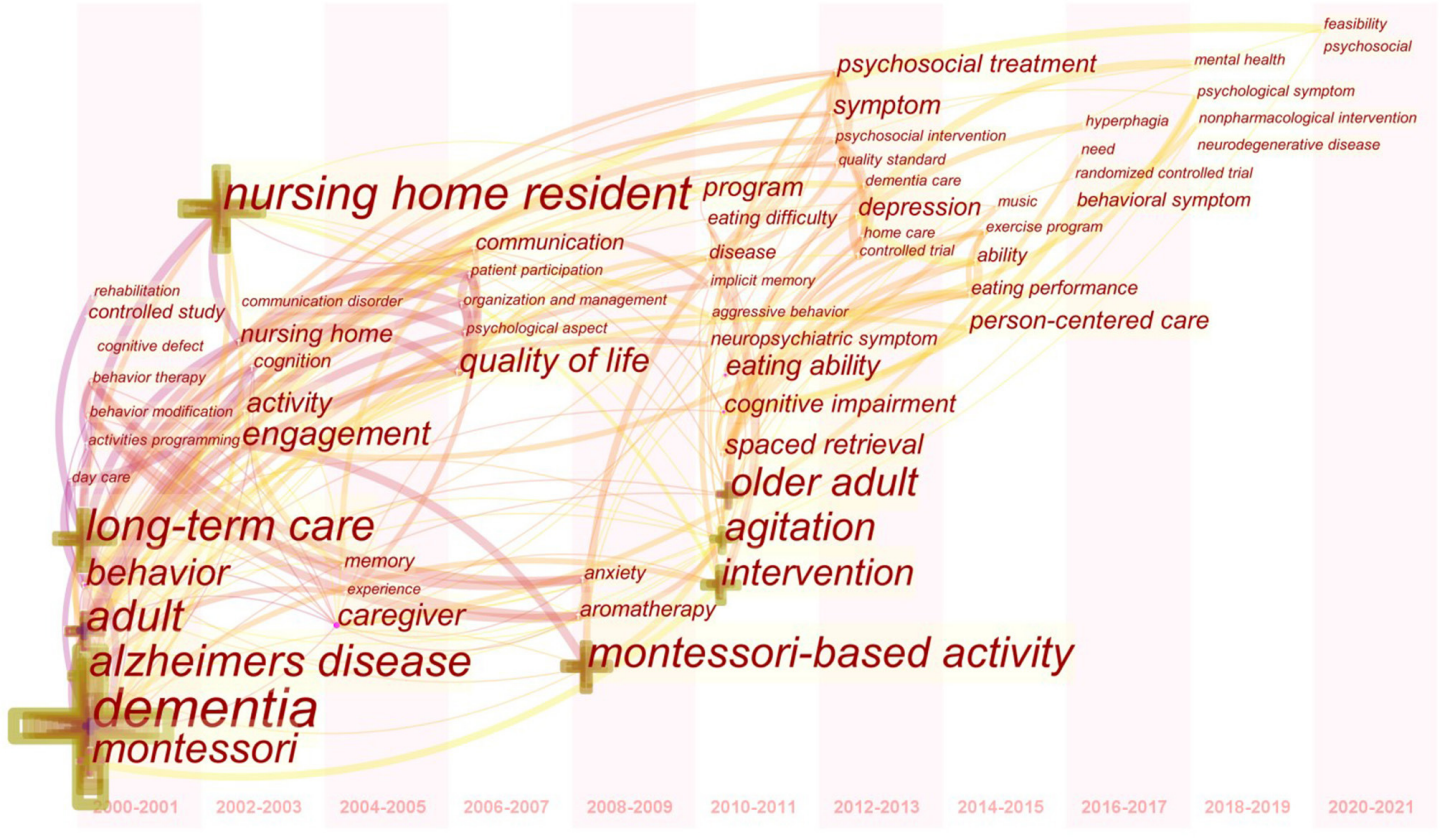
Time zone graph of keywords in articles published in English on Montessori approach to interventions for patients with dementia from 2000 to 2021.

In CiteSpace, the keywords were used as nodes for analysis, and the Timezone View function was selected in the Layout interface to distribute the keywords according to time. Figure 9 shows the distribution of keywords according to time in Chinese language publications. The research on the Montessori method of intervention in dementia patients in China started late, and in recent years, more attention has been paid to the eating ability of dementia patients and the aggressive behavior of dementia patients to improve the quality of survival. Figure 10 shows the distribution of keywords according to time in English language publications. Many researchers have started to pay attention to the behavioral problems of dementia patients from the early stages. As time goes by, the research has become more in-depth, focusing not only on the behavioral problems of dementia patients, but also on the psychosocial problems of dementia patients, promoting the physical and mental health of dementia patients and improving their psychosocial support.

### Analysis of Article Sources

In CiteSpace, source was used as nodes for analysis. The journal sources of Chinese language publications and English language publications were analyzed, as shown in Tables 1, 2. There were 21 sources of articles published in Chinese, among which only *Chinese Journal of Practical Neurological Diseases* and *Chinese Journal of Gerontology* contained 2 articles, and the rest of the journals contained 1 article. There were 65 sources of articles published in English, among which the journals contained above five articles were *Gerontologist, Dementia, International Psychogeriatrics, BMC Geriatrics, Journals of the United States of America*, and *International Journal of Geriatrics*. English language publication sources were more than Chinese language publication sources. The number of English journals which contained above two articles was higher than that of Chinese journals. Compared with the journals published in English language articles, the quality of journals published in Chinese language articles needs to be improved. Therefore, it can be inferred that the research of Montessori interventions in dementia patients have not yet formed a core group of journals in China.

**Table 1:** Top 2 source of journals published in Chinese language.

**Number**	**Source**	**Count**	**Percent**	**Journal source**
1	Chinese Journal of Gerontology	2	8.7%	Overview of Chinese core journals; Chemical Abstracts (CA); Japan Science and Technology Agency (JST)
2	Chinese Journal of Practical Nervous Diseases	2	8.7%	Japan Science and Technology Agency (JST)

**Table 2:** Top 5 source of journals published in English language.

**Number**	**Source**	**Count**	**Percent**	**Impact factor**
1	Gerontologist	8	7%	5.271
2	Dementia	7	6.2%	2.764
3	International Psychogeriatrics	7	6.2%	3.878
4	BMC Geriatrics	5	4.4%	3.921
5	Journal of the American Medical Directors Association	5	4.4%	4.669

## Discussion

This study uses the articles in the field of Montessori method interventions for dementia patients in the past 21 years collected in China National Knowledge Infrastructure, Wanfang, China Science and Technology Journal Database, the Web of Science core collection database, PubMed, and the Scopus database as the data source. We used Microsoft Excel, COOC software, and CiteSpace to analyze the data, as well as to organize and map the articles. We systematically analyzed the Chinese language publications and English language publications in this field, and through visual analysis of the map, we could clearly see the current status and hot spots of research in this field.

Research in China on Montessori methods for interventions in patients with dementia started in 2016, while international research in this area has been conducted by many researchers (31–34) since 2000. In terms of the number of publications, the overall trend of English language publications is on the rise, while the overall number of Chinese language publications is low. The United States is the country with the largest number of publications, and also has exchanges and cooperation with other countries, and has a greater influence. There are many issuing institutions both in China and abroad, mostly concentrated in universities, but the distribution of issuing institutions in China is relatively scattered, and only a few cooperative networks have been formed. By analyzing the sources of the articles, it was found that the sources of Chinese language publications were less than those of English language publications. Few journals contained more than two Chinese language publications. The reason for the paucity of Chinese language publications on Montessori interventions in dementia care may be that the attention to this field only started in recent years in China. In China, 94–99% of dementia patients are cared for by their families and research on related interventions is still immature (35). Chinese guidelines recommend that the first treatment considered for psycho-behavioral symptoms of dementia is non-pharmacological intervention. For severe psychotic-like symptoms of dementia, low-dose atypical antipsychotic treatment is available (36). However, it is difficult to care for patients with dementia, and the application of the Montessori approach to dementia patients in China is still being refined. Therefore, China should strengthen its research in this field and pay attention to exchange and cooperation with international institutions, so as to improve its influence on this field.

The keywords were analyzed and a keyword co-occurrence graph, burst graph, and time zone graph were produced using CiteSpace, which can visualize the current status of research, research hotspots, and development trends in the field of Montessori method interventions for dementia patients. Taken together, Chinese and international studies have focused on patients with Alzheimer's disease, aiming to use activities based on the Montessori method to improve patients' psycho-behavioral problems and improve their quality of life. In China, dementia is the fourth leading killer of elderly people after heart disease, cancer, and stroke, and there are no drugs or means to completely cure it (37). Dementia can bring a heavy mental as well as economic burden to patients and their families and is a serious social problem. Psycho-behavioral problems are a common symptom of dementia patients (3), and patients often suffer from physical and verbal aggression, wandering, hallucinations, and sleep disturbances (38), which seriously threaten their health. A large number of scholars abroad have already paid attention to patients' mental behavior problems, and research on this problem has only begun in China; therefore, scholars in China can refer to more mature studies abroad and design systematic and specific activities for dementia patients based on the Montessori method, so as to improve the quality of survival of dementia patients.

Psychosocial problems in dementia patients have been studied by international scholars who found loneliness and social isolation in dementia patients. Victor et al. (39) found that 30.1% of 1,547 dementia patients reported feeling moderately lonely. Loneliness is also a risk factor for psychiatric behavioral problems in patients with dementia (40). Chinese scholars have also conducted studies on the psychological problems of dementia patients and their caregivers (41, 42), but have not yet intervened using the Montessori approach. Therefore, our scholars should pay attention to the psychological situation of dementia patients along with their psycho-behavioral symptoms, which can be based on the Montessori approach to promote their physical and mental health.

By comparing the keyword burstness graphs and time zone graphs of Chinese language publications and English language publications, we can see that research on the Montessori approach to intervene in the eating ability of dementia patients started in 2017 in China. The eating ability in patients with dementia has been reported in English language publications as early as 2010. It is evident that the eating ability of patients with dementia is also a key concern for researchers. Difficulty in eating is a common problem in patients with Alzheimer's disease. More than 50% of patients with Alzheimer's disease face varying degrees of difficulty in eating (43), which can have a serious negative impact on the patients and caregivers (44). Impaired eating ability due to cognitive decline is an important factor in eating difficulties, especially for patients with moderate dementia (45). Referring to Camp's study, Wu et al. (46) designed activities based on the Montessori method to assess patients' eating abilities. They trained patients with dementia on eating behaviors, such as training them to pick up familiar foods. After 6 months of follow-up, the patients' eating difficulties were reduced. It was reported that a quiet and prepared eating environment kept the dementia patient in a good mood and allowed them to focus on eating. That was good for reducing aggressive behavior and improving the eating ability (47). Therefore, it is important to provide a quiet environment in the care setting and intervene in the patients' eating behavior based on the Montessori approach. It not only improves the patients' ability to eat, but also reduces the burden on the caregivers.

International scholars began to focus on caregivers of patients with dementia early on, and they promoted the participation of patients with dementia in activities by facilitating family-patient interactions. Patients with dementia are closer to their own families than they are to researchers, and they are more receptive to the behaviors and words of their families. In a study by Volland and Fisher (48), to enable Montessori activities for patients with dementia in a clinic setting, they left blank pages on a clipboard that allowed the family to guide the patient in drawing while waiting for an appointment. In this area, no studies have been conducted in China on caregivers of patients with dementia, and interventions have been done only at the patient level. Therefore, Chinese research can try to involve patients and caregivers in activities together, thus reducing patient resistance and promoting patient motivation to participate in activities.

The Montessori approach has been used for many years and it is scientific. In contrast to other non-pharmacological interventions, the Montessori approach emphasizes an individual-centered approach that engages the patients with teaching aids in a natural and prepared environment, allowing the patient to actively explore and learn. Many dementia patients with BPSD or eating problems have improved from Montessori-based activities. Family caregivers have taken on the task of caring for patients with dementia. However, it is difficult to care for dementia patients, especially BPSD patients. The Montessori approach can be extended to family caregivers. Through caregivers' progressive training, not only can the quality of life of patients be improved, but also the burden of caregivers can be reduced.

### Strengths and Limitations

The strengths of this study are as follows. First, this study is the first visual analysis report of the articles on the Montessori method of interventions for dementia patients in the last 21 years by applying CiteSpace, and a comparative analysis of Chinese language publications and English language publications was conducted. Second, international research in this field has been more mature, but in China it is yet to be developed, and this study can provide direction and reference for Chinese research in this field. However, this study is not without its limitations. In this study, six databases were searched, but the number of articles included in the databases was limited, and the number of retrieved articles was small and not comprehensive enough. This study only included three Chinese databases and three English databases. It was impossible to include all the articles about Montessori approach for interventions in patients with dementia. Therefore, this study chose to analyze Chinese-language articles and English-language articles about Montessori approach for interventions in patients with dementia from 2000 to 2021. However, as the retrieved literature contains many journals, the quality of some articles needs to be improved.

## Conclusions

This study provides a comparative analysis of research hotspots and trends in Chinese language publications and English language publications on Montessori interventions in dementia patients. The depth of research on Montessori intervention in dementia patients in China needs to be further expanded. Chinese research should draw on the hot spots and frontiers of research in English language publications and consider them in the context of our national situation to explore a Montessori intervention system suitable for dementia patients in China.

## Data Availability Statement

The raw data supporting the conclusions of this article will be made available by the authors, without undue reservation.

## Author Contributions

TZ was responsible for drafting the manuscript, as well as the acquisition, analysis, and interpretation of data. JQ collected, analyzed, and interpreted the data. HS and MX performed the literature search and in the development of the manuscript. YS contributed to the conception and design of the current study. YL provided professional advice and performed much of the editing of the manuscript. All authors read and approved the final manuscript.

## Conflict of Interest

The authors declare that the research was conducted in the absence of any commercial or financial relationships that could be construed as a potential conflict of interest.

## Publisher's Note

All claims expressed in this article are solely those of the authors and do not necessarily represent those of their affiliated organizations, or those of the publisher, the editors and the reviewers. Any product that may be evaluated in this article, or claim that may be made by its manufacturer, is not guaranteed or endorsed by the publisher.
